# Floral Temperature and Optimal Foraging: Is Heat a Feasible Floral Reward for Pollinators?

**DOI:** 10.1371/journal.pone.0002007

**Published:** 2008-04-23

**Authors:** Sean A. Rands, Heather M. Whitney

**Affiliations:** 1 Centre for Behavioural Biology, School of Clinical Veterinary Science, University of Bristol, Bristol, United Kingdom; 2 Department of Plant Sciences, University of Cambridge, Cambridge, United Kingdom; University of Utah, United States of America

## Abstract

As well as nutritional rewards, some plants also reward ectothermic pollinators with warmth. Bumble bees have some control over their temperature, but have been shown to forage at warmer flowers when given a choice, suggesting that there is some advantage to them of foraging at warm flowers (such as reducing the energy required to raise their body to flight temperature before leaving the flower). We describe a model that considers how a heat reward affects the foraging behaviour in a thermogenic central-place forager (such as a bumble bee). We show that although the pollinator should spend a longer time on individual flowers if they are warm, the increase in total visit time is likely to be small. The pollinator's net rate of energy gain will be increased by landing on warmer flowers. Therefore, if a plant provides a heat reward, it could reduce the amount of nectar it produces, whilst still providing its pollinator with the same net rate of gain. We suggest how heat rewards may link with plant life history strategies.

## Introduction

For reproduction, flowering plants rely on a wide range of pollinators, and employ a wide variety of tactics to attract them. In plant-pollinator mutualisms, the pollinators are typically rewarded for their visit–usually with carbohydrate-rich nectar, although other nutritional rewards such as pollen or wax may also be offered [1]–[3]. Many of these pollinators are ectotherms, and are sensitive to changes in environmental conditions such as temperature, which might present problems to plants in colder climates–how can a plant attract any ectothermic pollinators when the environment is too cold for them to travel between flowers? However, some of these ectothermic pollinators, such as bumble bees (*Bombus* spp.), are able to control their body temperature to allow them some independence from the environmental conditions [4], allowing them to operate in environments that would otherwise be too cold [5]. Bumble bees are thermogenic, and can actively increase their body temperature to the level necessary for flight [4], [6]–[8]. This active warming adds an extra energetic cost to foraging, where the amount of energy spent depends upon how much heat the bee loses through passive cooling before it starts to actively warm itself in preparation for flight. Therefore, the thermal environment the bee forages in may be important in determining its behaviour.

Bumble bees actively choose to forage at warmer artificial flowers, given a choice of warm or cold flowers yielding the same nutritional reward [9]. Flowers are capable of both actively producing heat [10], [11], and maximising heat collection and retention [12], [13], and heat has been shown to be a floral reward in species that do not produce nectar [14]–[16]. It is therefore feasible that nectar-producing plants could also use elevated floral temperature as an additional reward to make them more attractive to nectar-collecting pollinators. In this paper, we ask whether elevated floral temperatures are a feasible reward, using a foraging model that includes a heat reward for the visiting pollinator, as well as a direct energetic reward. Economic models have been constructed to predict the foraging behaviour of bees in response to various environmental parameters [17]–[23], but that which we present here is the first to consider the potential effects of floral temperature in influencing the foraging behaviour of bees.

**Figure 1:**
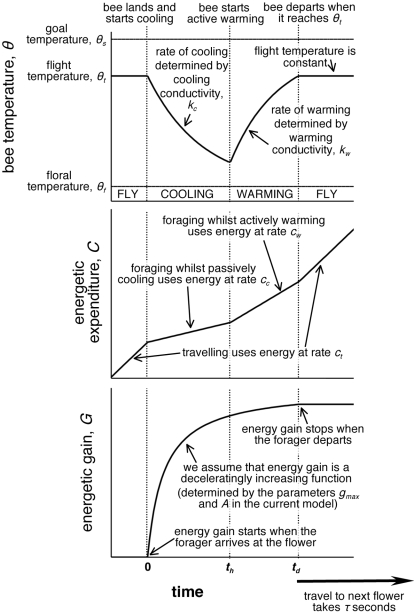
Sketch diagram of the model's components.

The model we present (sketched in figure 1) considers the effects of floral temperature on net gain rate. When a bee arrives at a flower, the gross amount of energy it collects follows a decelerating gain curve (as is standard in patch foraging models [24]), whilst its gross energetic expenditure is related to the costs of travelling, foraging without actively warming itself (which we call ‘passive cooling’), and foraging whilst actively warming itself (‘active warming’). When the bee is flying, it maintains an active flight temperature, and we assume that it has to reach this temperature in order to be able to leave the flower. On arrival, the bee passively cools until a point at which it decides to begins to actively warm itself in preparation for flight. The rate at which the bee cools is determined by the temperature of the floral environment, and therefore floral temperature can potentially play a large part in determining the visit length and net energetic gain of the pollinator. In this paper, we consider the effects of floral temperature and these other parameters on the behaviour of the pollinator, and we discuss how these might affect plant life history strategies.

## Results and Discussion

All the results described here are specific to parameters derived for bumble bees, as described in the methods section. The general form of the model suggests that the exact values of all the parameters used could have very large effects upon the qualitative predictions we can make about the visiting behaviour of pollinators to heated flowers. However, the sensitivity analyses conducted (described in the methods section, and presented in the supplementary material, figures S1, S2, S3, S4) demonstrate that the qualitative trends described below are robust for bumble bees.

**Figure 2:**
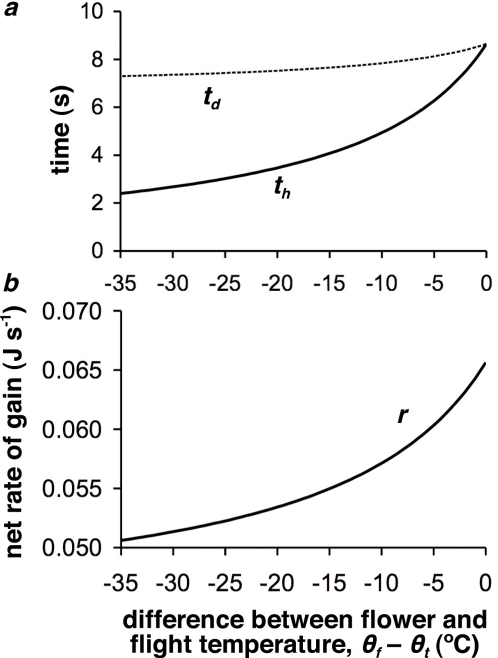
Effect of floral temperature on visit timing and gain rate. Showing effect on *a*) the time at which active heating begins, *t_h_*, and the departure time *t_d_*, and *b*) gain rate, *r*. Parameters as described in the methods section, but with θ*_f_* systematically adjusted.

From the model presented here, we would predict that the optimal length of time that the bee should spend on a flower will increase as the temperature of the flower gets closer to the body temperature necessary for flight (although the actual increase seen is small–figure 2a), if a bee is behaving in a manner that maximises the net rate of energy delivery to its nest. This is seen, for example, in the mining bee *Andrena bicolor*, which showed a positive correlation between visit length and floral temperature when visiting the solar-heated flowers of *Narcissus longispathus*, an early-flowering montane species [25]. Coupled with this increase in visit length with temperature is an increase in the net rate of gain (figure 2b), suggesting that providing warmth for pollinators could be the equivalent of the plant providing an extra metabolic reward. In the model we are assuming that the only point at which the bee stops flying is in flowers, and therefore the model does not consider other non-floral environmental temperatures, which are combined into the catch-all net cost of travelling term *c_t_*. This suggests that bees should spend longer per flower visit if the flower is warm (due to an increase in θ*_f_*), but less time in a flower if the costs of travelling are reduced (a reduction in *c_t_*), such as through an increase in the temperature of the extra-floral environment through which the bee has to travel.

Table 1 details other effects we would expect from changing model parameters. As would be predicted by the marginal value theorem [26], if the time spent travelling is small, the bee should leave the flower without collecting much nectar. As the distance between flowers increases, the bee has to expend considerably more energy in both reheating itself and fuelling the longer flights. These results suggest that in cold environments (where the temperature of flowers is at least that seen in the surrounding environment), bees will only be able to forage if many flowers are available within short distances (favouring a life history where plants are gregarious, common, and flower synchronously), or if rarer plants are available that provide a suitably high heat reward.

**Table 1:** Effects of model parameters on visit length *t_d_*.

variable	description	effects of increasing variable on visit length, *t_d_*
*A*	gain curve shallowness	increases
*c_c_*	cost of foraging whilst passively cooling	decreases
*c_t_*	cost of travel	increases
*c_w_*	cost of foraging whilst actively warming	decreases
*g_max_*	maximum gain from flower	decreases
*k_c_*	cooling conductivity	decreases
*k_w_*	warming conductivity	increases
τ	travel time	increases
θ*_f_*	floral temperature	increases
θ*_s_*	asymptotic temperature	increases
θ*_t_*	travel temperature	decreases

The environment external to the floral micro-climate will indirectly affect all the model parameters, but its effects are probably most apparent in the net cost of travel, *c_t_*. Within the modelling framework, the bee can only be affected by non-floral temperatures when it is in flight, and we assume that the bee has to maintain its body at a flight temperature θ*_t_* during this entire period of contact. Increasing flight temperature leads to a reduction in visit length (table 1), but we'd argue that it is biologically unclear why a bee should reduce θ*_t_*. Increasing the net cost of travel *c_t_* means that the bee spends longer in a flower. This suggests that bees should spend a longer time at warm flowers in cold environments, but this should occur in order to reduce the amount of time spent in the colder, energetically expensive non-floral environment, rather than because the bee has to spend more time actively raising its body temperature in preparation for flight.

Environmental temperature also fluctuates throughout the day and the season, but we don't consider this form of variation in the model. Flowers that are actively thermogenic may provide a constant source of predictable warmth, such as that recorded in the sacred lotus *Nelumbo nucifera* [27], which could influence the behaviour of their pollinating beetles. Flowers that are passively thermogenic through processes such as heliotropism will nonetheless offer a thermal microenvironment that differs greatly from external environmental conditions. There may therefore be a optimal time of day for pollinators to forage, tracking diurnal temperature variations [e.g. 28], and so if a pollinator is behaving optimally, it will not only change its visit times and possibly energetic expenditures as suggested by the model, but may also schedule its behaviour to make best use of diurnal variation. If the pollinator has a range of plant species that it can visit during the day, we could, for example, see heated flowers being preferred during the colder periods of the day (such as around dawn or dusk). This would be of advantage to species that flower at colder times of the year, or grow in colder environments [13], [25], [29], where providing heat not only provides an increase in the rewards offered to attract pollinators, but also may be essential to maintain the presence of any pollinators within the environment. This is of particular importance when we consider that climate change is causing changes in the phenology and community biology of organisms [30]. Effects on plant-pollinator communities have already been noted [31], [32], and careful consideration should be made of the thermal ecology of plants that provide a heat reward if we are to fully understand how the their population ranges and those of their pollinators will change over the next few decades.

**Figure 3:**
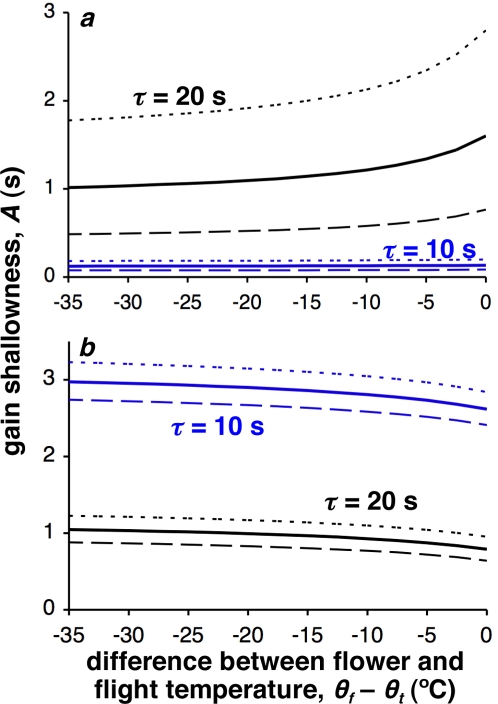
An increase in floral temperature means that nectar production can be reduced. *a*) Lines show paired values of floral temperature (shown as the difference between floral temperature and take-off temperature, θ*_t_*−θ*_f_*) and gain shallowness constant *A* (where a larger value means that it takes longer for the bee to collect a given amount of nectar), yielding the same optimal net rate of gain (for illustrative purposes, the lines represent the isocline where r* = 0.05 J s^−1^); *b*) temperature and gain shallowness pairs yielding the same optimal visit length, *t_d_* (for illustrative purposes, the lines represent the isocline where t_d_* = 7.5 s). In both figures, the solid black line uses the standard parameter set as described in the methods section, where travel time τ = 10 s. The solid blue line uses the same parameter set, but τ = 20 s. The dotted and dashed lines demonstrate a change in the cost of flight *c_t_* (standardised at 0.336 J s^−1^), representing one way in which non-floral environmental temperature can be included: the dotted lines use *c_t_* = 0.9×0.336 J s^−1^, and the dashed lines use 1.1×0.336 J s^−1^.

We can also make inferences about floral evolution from this model. If a bee's net energetic gain is influenced by its energetic expenditure, then a warm flower will reduce this expenditure: essentially, the value of a unit of nectar will increase if flower temperature is raised. From the results presented, we would predict that a plant could reduce nectar quality (e.g. the quantity of sugars put into a unit quantity of nectar) but still provide the same net rate of gain to a visiting pollinator (demonstrated in figure 3a). Nectar secretion is likely to decrease at low temperatures [33]–[35], and so floral warming may also be a mechanism by which the flower increases nectar production. It is therefore feasible that warmth could act as a cue (where ‘warmer flower’ signals ‘more nectar’) as well as a reward to the pollinator (although warmer flowers may allow the flower to cut its costs by producing less or lower quality nectar). Honey bees have been found to be able to use air temperature as a cue [36]. Temperature receptors, located in the bee's antennae, are acutely sensitive to temperature variations, and can sense differences of 0.25°C. With this degree of resolution in the air, it is feasible that bees would be able to display equal sensitivity to flower temperature.

Here, we choose to model nectar uptake by the bee with a Michaelis-Menten-like function, assuming that the bee experiences diminishing returns for longer stays in the flower (evidence suggests that a diminishing returns curve may well be appropriate [37]). We would argue that this gain function considered here is sufficient for the intentions of the model (although we argue in the methods section that the parameters used in the model can have large effects upon the direction of the trends described here, the sensitivity analyses, presented in figures S1 and S2 of the supporting information, demonstrate that the qualitative trends described are robust for the bee-specific parameters presented here). We also considered the case where nectar uptake follows a step-like function, which could occur where the pollinator is foraging on a compound ‘flower’ consisting of a platform of separate flowers (such as the individual flowers in the umbels of the Apiaceae), or where the pollinator is ingesting nectar in discretised units, such as in lapping groups like the Diptera [38]. Sensitivity analyses (presented in figures S3 and S4 of the supporting information) demonstrate that similar results are gained for a step-like function where there is a diminishing return rate with time spent at the flower.

Figure 3a demonstrates that, as average travel distance increases, nectar quality can be reduced by an increasingly large amount as floral temperature increases. Therefore, if plants are widely dispersed and provide a heat reward, they can reduce the quality of the nectar that they produce, and still compete with other cold flowers that produce high quality nectar (although figure 3b shows that if the plant increases its temperature and requires the pollinator to visit for a set length of time, it needs to increase the quality of the nectar in order to maintain the pollinator's visit length at higher temperatures: within species, nectar secretion has been shown to increase with increasing temperature [33]–[35], [39]). We have shown above that when a bee experiences an increase in floral temperature, it should increase its visit length. However, the corresponding change in the optimal net gain rate experienced by the bee isn't very large, as seen in the relatively flat line for the departure time *t_d_* in figure 2. This suggests that if a bee is maximising the net rate of energy delivery to its nest, there may be little difference between staying longer at a warm flower, compared to foraging at many cold flowers, if we made the large assumption that warm and cold flowers are otherwise similar in nectar quality and delivery (which could perhaps occur if there is phenotypic variation in the warming behaviour seen within a plant species).

In this plant-pollinator system, the pollinator faces a simple trade-off between temperature and nectar quality (both affecting its net energetic gain). For the plant, energetic costs are incurred in nectar production [40]–[42], whilst floral temperature regulation can be energetically expensive in some cases [10], but may also be passive through reflecting environmental heat [reviewed by 14]. Furthermore, nectar production occurs solely for the purpose of attracting pollinators, whilst floral heat has multiple roles, affecting plant development [13] as well as pollinator attraction. Heat production could therefore also have effects upon fitness that aren't mediated by pollinators, if it affects the quality and longevity of the pollen and nectar produced, or changes the plant's expenditure of resources on maintaining the floral tissues (which could be especially costly as thermogenic flowers tend to be large in order to retain heat, as noted in [43]). Because there are costs and benefits to heat production and regulation within flowers, we could explore optimal floral strategy using optimisation techniques, which may reveal that different species compete for pollinators using a variety of different rewards. It should also be remembered that visitors to warm flowers may outstay their welcome, as there is little benefit to the plant of a pollinator (or non-pollinator) remaining in the flower for any length of time longer than that sufficient to deposit/pick up pollen. The plant's strategy will therefore have been shaped by a variety of selective pressures and developmental constraints [3], and so environmental and life history constraints need to be considered before we can make predictions about the strategy of a particular species.

## Methods

### Cooling and heating processes

The following model considers a basic representation of the processes of temperature change within the bee [7]: apart from the processes highlighted below, we ignore other heat transfer through processes of conduction, convection and insolation. We assume that when the bee lands at the flower, it cools at a rate proportional to the difference between its current temperature θ and the temperature of the immediate floral micro-climate θ*_f_*, where
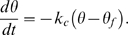
If we assume that the flying bee is at travelling temperature θ*_t_* when it arrives at the flower at *t* = 0 and that the cooling conductivity *k_c_*>0, then the bee's temperature at time *t* is 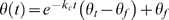. If the bee starts warming up again at time *t_h_*, when its temperature is θ*_h_*, then
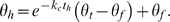
(1)Between *t_h_* and the point at which the bee leaves the flower, we assume that the rate of warming is proportional to the difference between its current temperature and a goal temperature θ*_s_*, where
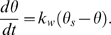
(2)For convenience, we assume that θ*_s_*>θ*_t_*, and the warming conductivity *k_w_*>0. Given the condition that the bee departs the flower at time *t_d_* when it has reached θ*_t_*, we can solve (2). Making the further assumption that the bee is at θ*_h_* at *t_h_*, we can substitute (1) into this expression to give
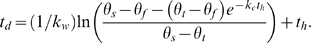
(3)It can be demonstrated that *dt_d_*/*dt_h_*>1 and *d* ^2^ *t_d_*/*dt_h_* ^2^<0 for biologically relevant (positive) values of *t_h_*, meaning that *t_d_* is a deceleratingly increasing function of *t_h_*.

### A general model of optimal floral visiting times

We assume that there are different net metabolic costs when the bee is simply foraging at a flower (when the bee is assumed to be cooling down to background temperatures), and when it is foraging and warming at the same time. These ‘cooling’ and ‘warming’ costs take *c_c_* and *c_w_* units of energy per unit time respectively. The costs must also take into account τ, the time taken travelling to and from the flower, and *c_t_*, the energetic cost of this travel: it is assumed that the bee maintains its body temperature at θ*_t_* during flight, and that this cost takes into account the fact that the extra-floral environment through which the bee travels will be colder than the flower, where the heat transfer described above took place. We note here that *c_t_* isn't just the cost of flight, but rather it represents the net metabolic cost of the bee when it is in flight. For simplicity, we assume that the bee is not able change its flight speed, or energetic expenditure during flight in response to fluctuating environmental conditions. The total energetic cost of a visit of length *t_d_* is therefore
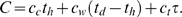
(4)


When the bee is foraging at a flower, we assume that it gains energy, but energy gain occurs at rate of diminishing returns curve (as is discussed in [37]) with respect to the length of time spent on the flower, *t_d_*. Consequently, it is possible to demonstrate that *G* is a deceleratingly increasing function of *t_h_* (where 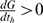 and 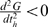, given that *t_d_* is an deceleratingly increasing function of *t_h_* as described above).

The bee's net gain during a visit of length *t_d_* is expressed as 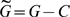.
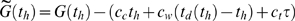
(5)The journey time that maximises overall gain rate can be found using the techniques used to derive the Marginal Value Theorem [26]. If the rate of gain for a bee that spends *t_d_* in a flower is 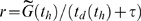, the bee optimises its net energy gain rate when *dr*/*dt_h_* = 0 (and second order conditions for a maximum are satisfied). This generates a transcendental relationship, solved here using computational techniques. Note that this model is specific to thermogenic central-place foragers that rest within the flower to gain heat (specifically, bumble bees), and is not suitable for predicting the behaviour of hovering foragers that don't enter the flower's microclimate (such as hawk moths, hummingbirds or bats).

Differentiating with respect to *t_h_*,
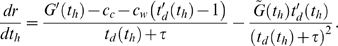
(6)The rate of gain is optimised when *dr*/*dt_h_* = 0 and second order conditions are met, which occurs at t_h_*. Setting (6) to zero, we rearrange to give an expression for 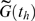 when *t_h_* = t_h_*. Substituting into (5), we find that
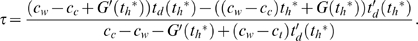
(7)This equation demonstrates that unless we know the exact forms of *G*(*t_h_*) and *t_d_*(*t_h_*), we cannot make clear predictions about whether t_h_* should increase or decrease with respect to an increase in τ, the journey time between flowers. Similarly, by rearranging (7), we are also unable to make clear predictions about changes in t_h_* with respect to changes in *c_c_*, *c_t_* and *c_w_*, the metabolic costs.

The second derivative of *r* with respect to *t_h_* is
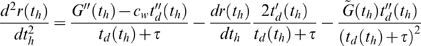
At t_h_*, the middle term is equal to zero. Therefore, the second derivative is negative if
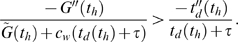
It has already been stated that *G*″(*t_h_*) and *t*″*_d_*(*t_h_*) take negative values in the region of biological interest, and we assume that the bee will only forage if the net gain is positive (so 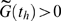). Whether the stationary value found when *dr(t_h_)*/*dt_h_* is a maximum or minimum therefore depends upon the exact shape of the *G* and *t_d_* functions.

### An example specific to bumble bee foraging

Here, we assume that the gain function *G* takes a Michaelis-Menten form with respect to the time spent on the flower:
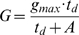
(8)where *g_max_* is the maximum amount of energy that can be gained from a flower in a visit, and *A* is an arbitrary time constant. This form of the gain function was used within the framework described above to explore a bumble bee-specific model. Parameter values for foraging bumble bees were estimated to be *k_w_* = 0.01 s^−1^, *k_c_* = 0.003 s^−1^, θ*_t_* = 35°C, θ*_s_* = 40°C (based upon the figures published in [6] and [44]), θ*_f_* = 20°C (chosen arbitrarily), *c_w_* = 0.121 J s^−1^ (estimated from [6], for a bee with a 0.143 g thorax), *c_c_* = 0.042 W (from [20]), and *c_t_* = 0.336 J s^−1^ (based on the assumption in [20], that *c_t_* is approximately eight times the cost of feeding, which we equate here to *c_c_*). Note that the *c_c_* used here is specific to honey bees, rather than bumble bees (for which we were unable to find suitable figures: the values of *c_c_*, *c_t_* and *c_w_* for bumble bees is likely to be higher due to their larger body size, but we assume that they will be proportionally similar to each other once they are scaled to accommodate this bumble bee size difference). We parameterised the curve describing nectar gain to give gain values of up to and around 10 J visit^−1^ (with *g_max_* = 10 J and *A* = 1 s), approximating the gain and time range used in [21].

Sensitivity analyses were conducted for these predicted parameters, examining the effects of increasing or decreasing the parameters by 50%. As demonstrated in the supporting information (figures S1 and S2), these changes had no effect upon the qualitative predictions made in the paper.

Using the same parameters as above, we also explored using a step-like gain function, which took the integer part of the Michaelis-Menten-like equation given in (8):
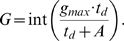
Again, we conducted sensitivity analyses to explore the effects of varying the bee-specific parameters by up to 50%. As demonstrated in the supporting information (figures S3 and S4), these changes had no effect upon the qualitative predictions made in the paper, although extreme values of gain shallowness constant *A* had some effect upon the trends seen.

## Supporting Information

Figure S1 Results with a Michaelis-Menten-like gain function considering variation in *k_w_*, *k_c_*, *c_c_*, *c_w_*, and *c_t_*. The graphs present the changes in the optimal value of *t_h_*, *t_d_* and *r* when floral temperature *θ_f_* is systematically altered. The five lines on each panel represent the optimal results for the parameter being changed (shown at the top left of each panel), where the parameter takes 50% (dotted line), 75%, 100% (thick line), 125% and 150% (dashed line) of the value given in the methods section.
(0.33 MB PDF)

Figure S2 Results with a Michaelis-Menten-like gain function considering variation in *τ*, *g_max_*, *A*, *θ_s_*, and *θ_t_*. The graphs present the changes in the optimal value of *t_h_*, *t_d_* and *r* when floral temperature *θ_f_* is systematically altered. The five lines on each panel represent the optimal results for the parameter being changed (shown at the top left of each panel), where the parameter takes 50% (dotted line), 75%, 100% (thick line), 125% and 150% (dashed line) of the value given in the methods section (with the exception of values for *θ_s_*, taken to be 37.5°C, 38.75°C, 40°C, 41.25°C and 42.5°C, and the values for *θ_t_*, taken to be 31°C, 33°C, 35°C, 37°C and 39°C). For the *g_max_* results, the 50% value gives too low a maximum gain to give calculable results and consequently isn't displayed.
(0.36 MB PDF)

Figure S3 Results with a step-like gain function considering variation in *k_w_*, *k_c_*, *c_c_*, *c_w_*, and *c_t_*. The graphs present the changes in the optimal value of *t_h_*, *t_d_* and *r* when floral temperature *θ_f_* is systematically altered. The five lines on each panel represent the optimal results for the parameter being changed (shown at the top left of each panel), where the parameter takes 50% (dotted line), 75%, 100% (thick line), 125% and 150% (dashed line) of the value given in the methods section.
(0.27 MB PDF)

Figure S4 Results with a step-like gain function considering variation in *τ*, *g_max_*, *A*, *θ_s_*, and *θ_t_*. The graphs present the changes in the optimal value of *t_h_*, *t_d_* and *r* when floral temperature *θ_f_* is systematically altered. The five lines on each panel represent the optimal results for the parameter being changed (shown at the top left of each panel), where the parameter takes 50% (dotted line), 75%, 100% (thick line), 125% and 150% (dashed line) of the value given in the methods section (with the exception of values for *θ_s_*, taken to be 37.5°C, 38.75°C, 40°C, 41.25°C and 42.5°C, and the values for *θ_t_*, taken to be 31°C, 33°C, 35°C, 37°C and 39°C). For the *g_max_* results, the 50% value gives too low a maximum gain to give calculable results and consequently isn't displayed.
(0.31 MB PDF)
