# Supplementary figures and images for: Floral Temperature and Optimal Foraging: Is Heat a Feasible Floral Reward for Pollinators?
Source: PLoS One. 2008 Apr 23;3(4):e2007. doi: 10.1371/journal.pone.0002007 (PMC2292243; doi:10.1371/journal.pone.0002007)

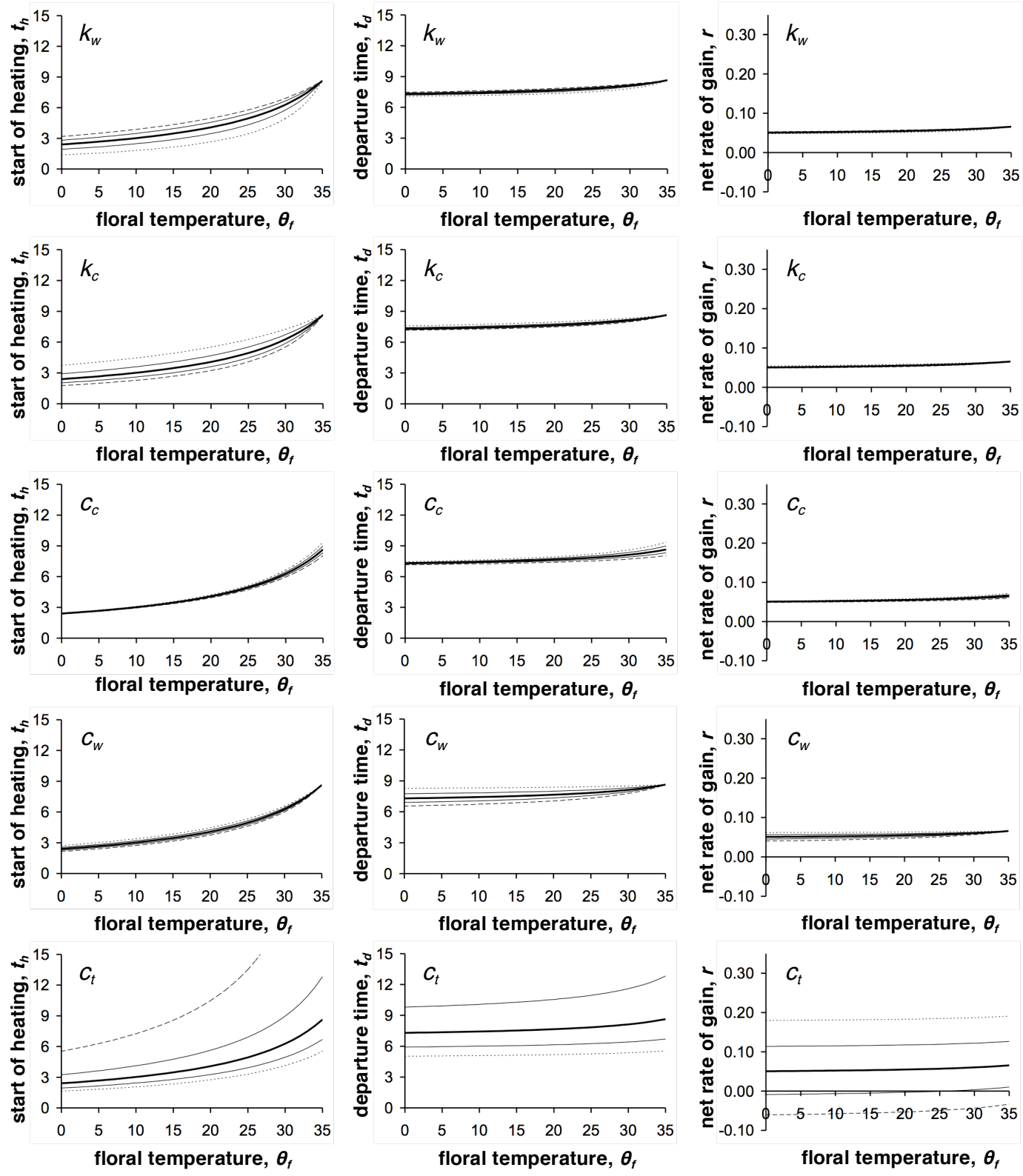

Supplement: Figure S1 — Results with a Michaelis-Menten-like gain function considering variation in kw, kc, cc, cw, and ct. The graphs present the changes in the optimal value of th, td and r when floral temperature θf is systematically altered. The five lines on each panel represent the optimal results for the parameter being changed (shown at the top left of each panel), where the parameter takes 50% (dotted line), 75%, 100% (thick line), 125% and 150% (dashed line) of the value given in the methods section. (0.33 MB PDF) [file pone.0002007.s001.pdf]

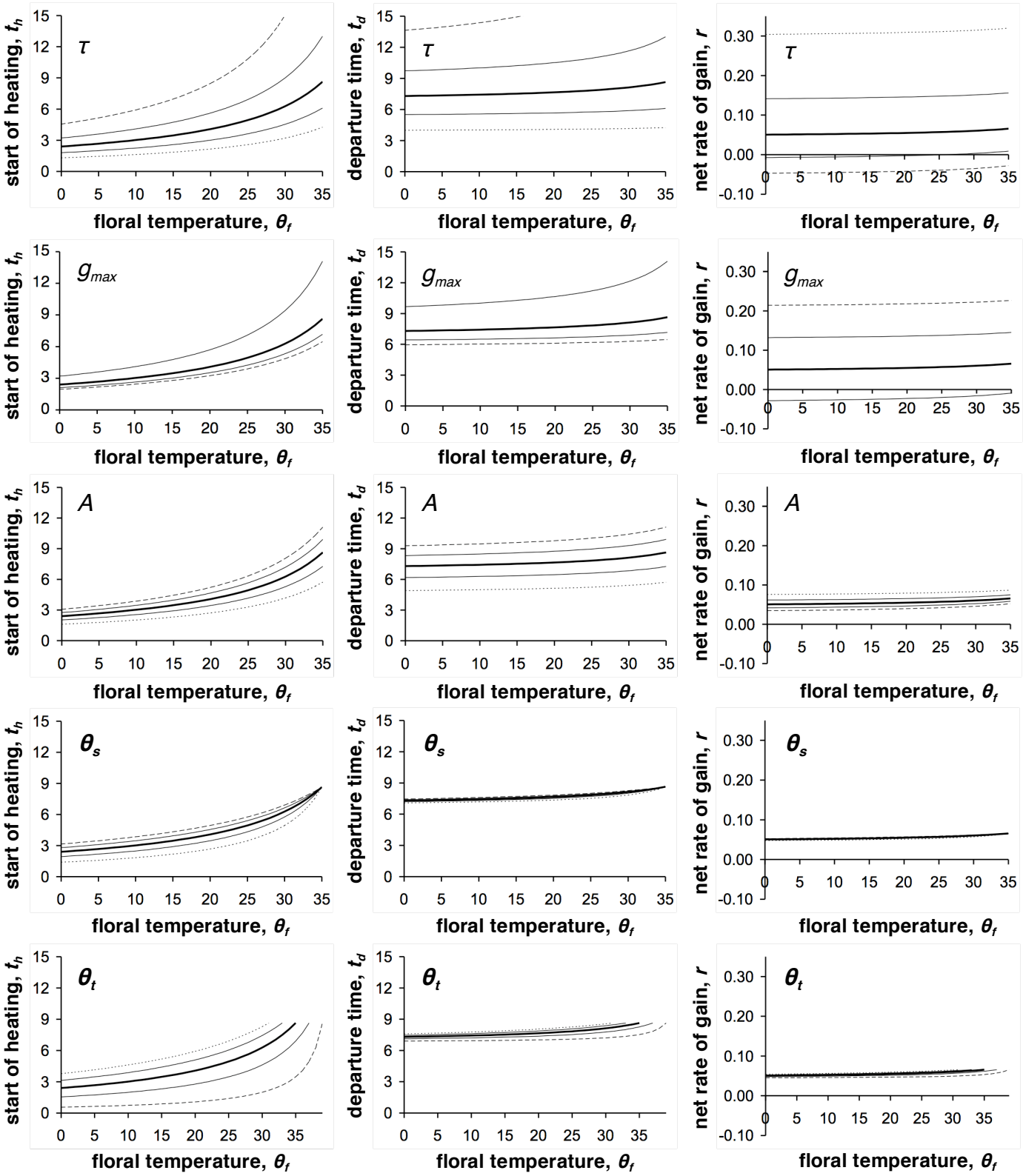

Supplement: Figure S2 — Results with a Michaelis-Menten-like gain function considering variation in τ, gmax, A, θs, and θt. The graphs present the changes in the optimal value of th, td and r when floral temperature θf is systematically altered. The five lines on each panel represent the optimal results for the parameter being changed (shown at the top left of each panel), where the parameter takes 50% (dotted line), 75%, 100% (thick line), 125% and 150% (dashed line) of the value given in the methods section (with the exception of values for θs, taken to be 37.5°C, 38.75°C, 40°C, 41.25°C and 42.5°C, and the values for θt, taken to be 31°C, 33°C, 35°C, 37°C and 39°C). For the gmax results, the 50% value gives too low a maximum gain to give calculable results and consequently isn't displayed. (0.36 MB PDF) [file pone.0002007.s002.pdf]

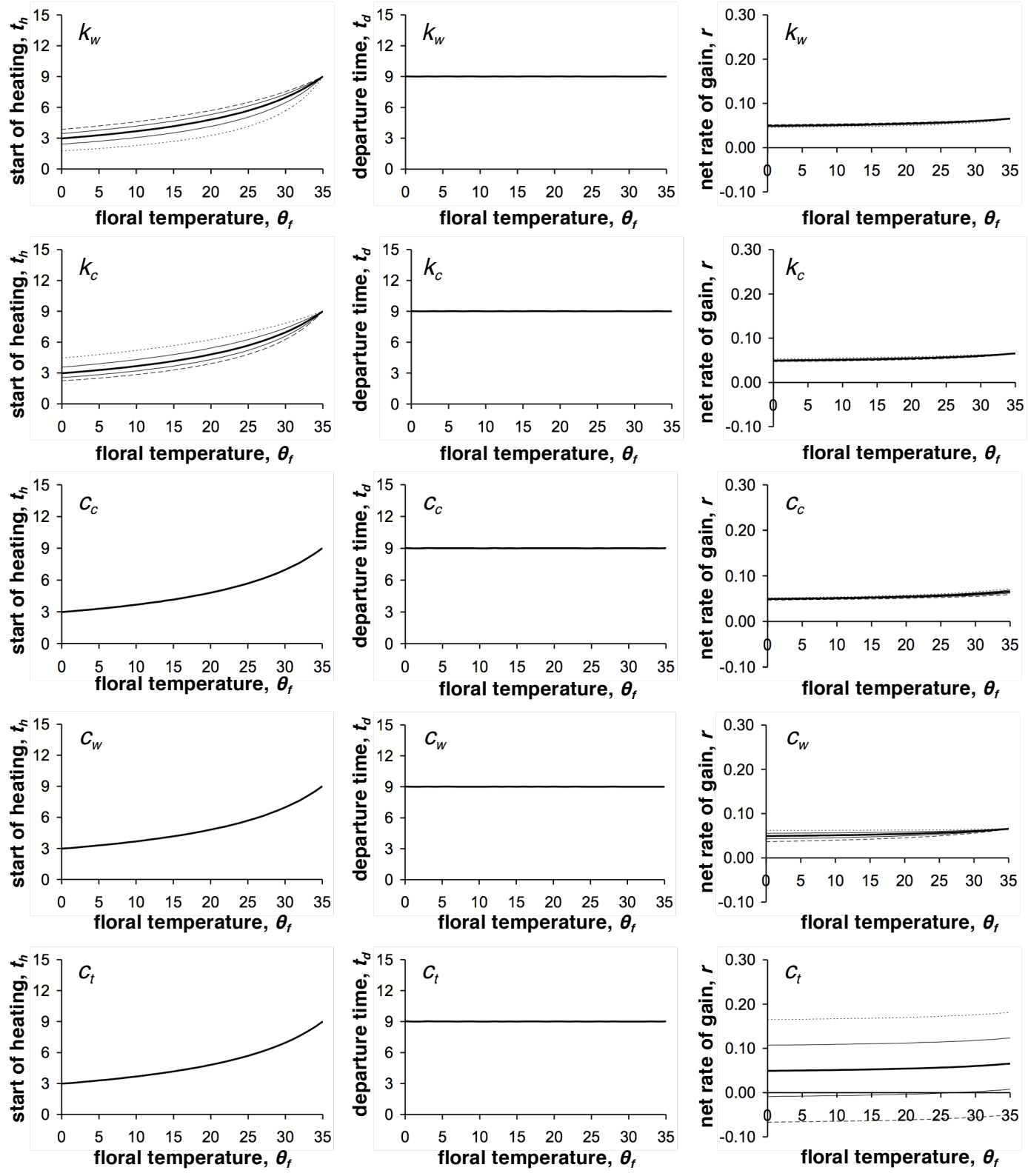

Supplement: Figure S3 — Results with a step-like gain function considering variation in kw, kc, cc, cw, and ct. The graphs present the changes in the optimal value of th, td and r when floral temperature θf is systematically altered. The five lines on each panel represent the optimal results for the parameter being changed (shown at the top left of each panel), where the parameter takes 50% (dotted line), 75%, 100% (thick line), 125% and 150% (dashed line) of the value given in the methods section. (0.27 MB PDF) [file pone.0002007.s003.pdf]

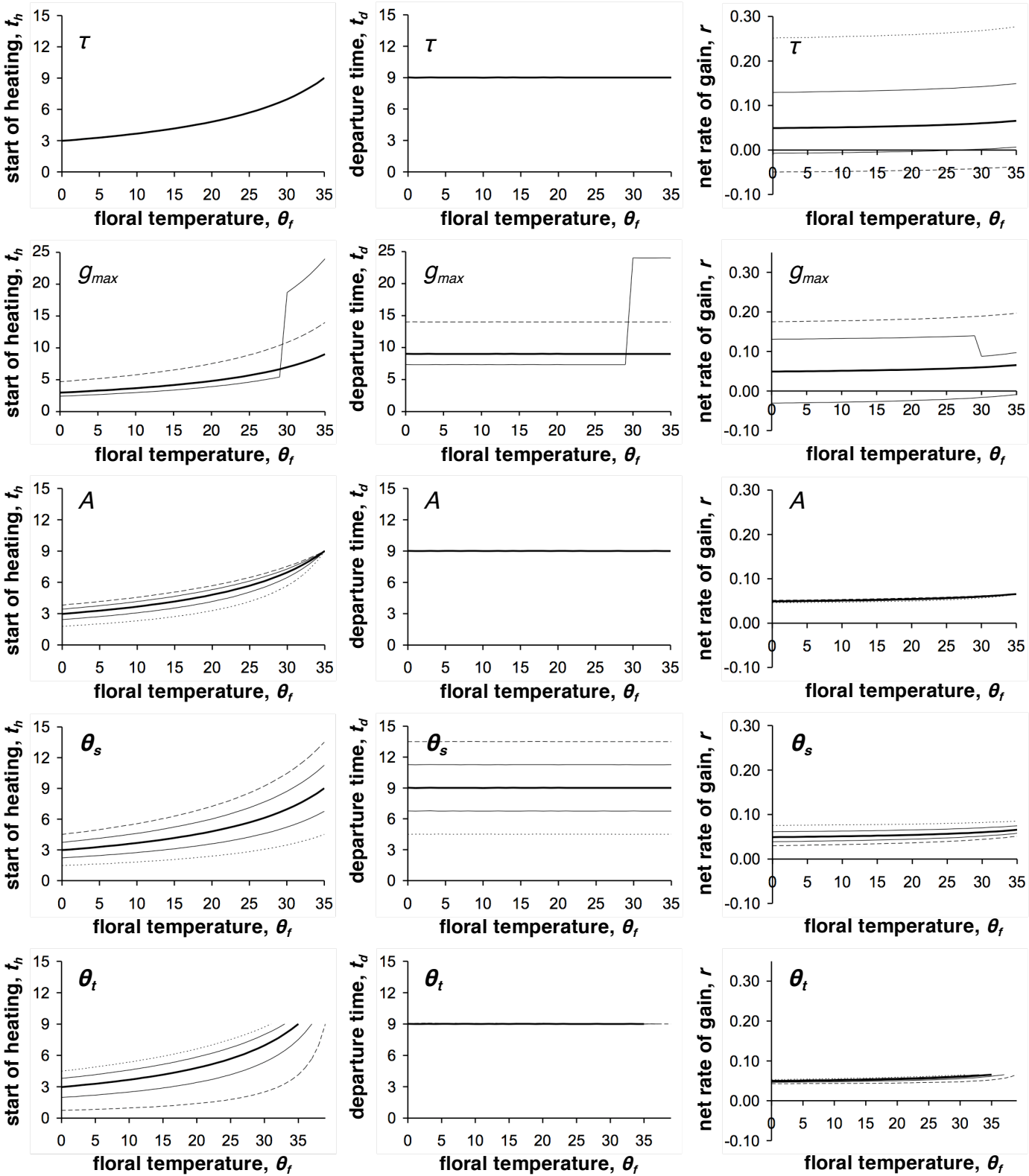

Supplement: Figure S4 — Results with a step-like gain function considering variation in τ, gmax, A, θs, and θt. The graphs present the changes in the optimal value of th, td and r when floral temperature θf is systematically altered. The five lines on each panel represent the optimal results for the parameter being changed (shown at the top left of each panel), where the parameter takes 50% (dotted line), 75%, 100% (thick line), 125% and 150% (dashed line) of the value given in the methods section (with the exception of values for θs, taken to be 37.5°C, 38.75°C, 40°C, 41.25°C and 42.5°C, and the values for θt, taken to be 31°C, 33°C, 35°C, 37°C and 39°C). For the gmax results, the 50% value gives too low a maximum gain to give calculable results and consequently isn't displayed. (0.31 MB PDF) [file pone.0002007.s004.pdf]
